# The Effect of Melatonin on Locomotor Behavior and Muscle Physiology in the Sea Cucumber *Apostichopus japonicus*

**DOI:** 10.3389/fphys.2019.00221

**Published:** 2019-03-19

**Authors:** Kui Ding, Libin Zhang, Tao Zhang, Hongsheng Yang, Richard Brinkman

**Affiliations:** ^1^CAS Key Laboratory of Marine Ecology and Environmental Sciences, Institute of Oceanology, Chinese Academy of Sciences, Qingdao, China; ^2^Laboratory for Marine Ecology and Environmental Sciences, Qingdao National Laboratory for Marine Science and Technology, Qingdao, China; ^3^University of Chinese Academy of Sciences, Beijing, China; ^4^Center for Ocean Mega-Science, Chinese Academy of Sciences, Qingdao, China; ^5^Australian Institute of Marine Science, Townsville, QLD, Australia

**Keywords:** melatonin, sea cucumber, locomotor behavior, physiological mechanism, fatty acid oxidation, oxidative phosphorylation

## Abstract

Melatonin is a highly conserved hormone in evolutionary history. It occurs in numerous organisms and plays a role in the endocrine and immune systems. Locomotor behavior is a basic behavior in animals and is an important indicator of circadian rhythms, which are coordinated by the nervous and endocrine systems. To date, the effect of melatonin on locomotor behavior has been studied in vertebrates, including syrian hamsters, sparrows, rats, zebrafish, goldfish, and flatworms. However, there have been few studies of the effects of melatonin on locomotor behavior in marine invertebrates. The goals of present study were to show the existence of melatonin in the sea cucumber *Apostichopus japonicus* and to evaluate its effect on locomotor activity. In addition, muscle tissues from control and melatonin-treated sea cucumbers were tested using ultra performance liquid chromatography and quadrupole time-of-flight mass spectrometry (UPLC-Q-TOF-MS) to determine the changes of metabolic activity in muscle. Melatonin was present in the coelomic fluid of *A. japonicus* at a concentration of ∼135.0 ng/L. The total distance traveled and number steps taken over 9 h after melatonin administration decreased with increasing concentration of the melatonin dose. Mean and maximum velocity of movement and stride length and stride frequency also decreased, but their differences were not statistically significant. Overall, these results suggest that melatonin administration had a sedative effect on *A. japonicus*. The levels of 22 different metabolites were altered in the muscle tissues of melatonin-treated sea cucumbers. Serotonin, 9-*cis* retinoic acid, all-trans retinoic acid, flavin mononucleotide in muscles were downregulated after melatonin administration. Moreover, a high free fatty acid (FFA) concentration and a decrease in the adenosine 5′-triphosphate (ATP) concentration in the muscle tissues of the melatonin-treated group were detected as well. These results suggest that the sedative effect of melatonin involves some other metabolic pathways, and the reduced locomotor modulator—serotonin, inhibited fatty acid oxidation and disturbed oxidative phosphorylation are potential physiological mechanisms that result in the inhibitory effect of melatonin on locomotion in sea cucumbers.

## Introduction

In 1958, melatonin was discovered from the pineal gland of cows (Lerner et al., 1958), and it subsequently was confirmed to be an indole neuroendocrine hormone (Reiter, 1995). This discovery triggered studies of the characteristics, physiology, medical applications, and behavioral functions of this special molecule. Melatonin is a highly conserved molecule in evolutionary history, and it occurs in organisms as different as plants and animals (Poeggeler, 1993). It is presumed to be a phylogenetically omnipresent hormone that primarily is involved in the regulation of circadian rhythm (Peres et al., 2014). Considering the extensive distribution of melatonin receptors in various tissues and organs of vertebrates, this hormone can play roles in diverse physiological processes (Zhdanova, 2005; Kulczykowska et al., 2006). In vertebrates, melatonin can be produced in the gastrointestinal system (Bubenik, 2002) and even the retina (Do Carmo Buonfiglio et al., 2011), but its main source of production is the pineal gland. In invertebrates, this hormone has been detected in numerous organs in insects (including the brain, palp, eyes, ovipositor, hind-leg, and ovary) and in the gonad of sea stars (Vivien-Roels and Pévet, 1993; Itoh et al., 1995; Peres et al., 2014). Melatonin also has been discovered in many other non-vertebrate organisms, including plants (Kolár and Machácková, 2005), dinoflagellates (Balzer and Hardeland, 1991), and fungi (Hardeland, 1999). However, the expression of melatonin in most invertebrates does not show obvious rhythmicity (Tanaka et al., 2007).

The effect of melatonin on locomotor behavior has been studied extensively in animals, especially in vertebrates. Melatonin has been shown to significantly reduce the locomotor activity in hamsters (Golombek et al., 1992) and have a sedative-like effect in cats, rats, and mice (Golombek et al., 1996). Locomotor inhibition by melatonin in the brain of rodents may be mediated by the central gamma aminobutyric acid (GABA) ergic system (Golombek et al., 1996). Similarly, it induces birds, fish and flatworms to fall asleep (Mintz et al., 1998; Zhdanova et al., 2008; Omond et al., 2017). Fewer studies of the effects of melatonin on locomotor behavior of invertebrates have been conducted. In a study of crickets, motor activity was reduced during the light-dark cycle as the melatonin concentration increased (Yamano et al., 2001). In another study of crayfish, melatonin had different effects on locomotor behavior depending on time of day of the melatonin injection: increased activity when treated during mid-photophase, no effect at late photophase, and decreased activity in early scotophase (Tilden et al., 2003a). Analogous results were reported for the fiddler crab *Uca pugilator*, which suggests that this feature is common in arthropods (Tilden et al., 2003b). Administration of exogenous melatonin also decreased locomotion rates in nematodes (Tanaka et al., 2007). However, the effect of melatonin on locomotor performance in other invertebrates, such as echinoderms, and how it might function remain unclear.

The sea cucumber *Apostichopus japonicus* is widely distributed along the shallow coast of northeastern Asia (Liao, 1980), and it is exploited as an important fishery resource in China, Japan, Korea, and Russia (Sloan, 1985). It also is a delicacy and a traditional medicine in China (Yang et al., 2015). As one of the most important benthic creatures in the marine environment in these areas, the activities of *A. japonicus*, including feeding, moving, and reproducing, may have a significant influence on the energy cycle of the marine ecosystem (Pan et al., 2015). The locomotor velocity of sea cucumbers is very slow (<3 mm/s) (Qiu et al., 2014; Pan et al., 2015). In addition, the coelomic cavity between the visceral mass and body wall is filled with coelomic fluid (Eliseikina and Magarlamov, 2002). Many types of coelomocytes are suspended in the coelomic fluid and may play important roles in the physiological processes of sea cucumbers (Eliseikina and Magarlamov, 2002). These features make sea cucumbers an ideal model system to investigate the behavioral endocrinology of marine invertebrates.

In living organisms, a complete and sophisticated regulation system and a complex metabolic network are involved in the production and regulation of the matter and energy that are necessary to support life. Most of the regulatory metabolites are small molecules that are present throughout the organism. Their concentrations are small, but their biological effects are powerful. These molecules are the physical basis for regulation of the neuroendocrine and immune systems in organisms. Metabolites are the final products that generated during living activities, and metabolomics analysis is study of these downstream molecules (Sharfstein et al., 2007). Metabolomics is an important tool for systems biology, as it provides the ability to measure the concentrations of endogenous small molecules in biofluids and characterize the quantitative changes of the metabolites that occur in specific physiological states of organisms (Nicholson et al., 1999; Sun et al., 2017). Ultraperformance liquid chromatography and quadrupole time-of-flight mass spectrometry (UPL-Q-TOF-MS) is a high resolution and sensitive metabolomics technology that can detect multiparametric metabolite profiles from biofluids or tissues rapidly and effectively (Wilson et al., 2005; O’Connor and Mortishire-Smith, 2006). This technology has been successfully applied to assess the metabolomic function of muscles in sea cucumbers between breeding and non-breeding stages (Ru et al., 2017), to study metabolic characteristics of intestinal fistula and obesity in humans (Yin et al., 2006; Kim et al., 2010), and to measure blood constituents in rats after treatment with traditional Chinese medicine (Wang et al., 2008, 2018).

The goals of the present study were to show the presence of melatonin in *A. japonicus*, evaluate its effect on locomotor behavior in this sea cucumber, and identify potential metabolic mechanisms involved in this effect. UPL-Q-TOF-MS metabolomic detection was performed on muscle tissues of sea cucumbers to identify the key metabolites and potential pathways that are involved in the effect of melatonin administration on locomotor activity. In order to verify the final effects of potentially changing signaling pathways on muscles, free fatty acid (FFA) and adenosine triphosphate (ATP) concentrations in the muscle tissue were measured as well.

## Materials and Methods

### Animals Rearing and Melatonin Detection

Sea cucumbers (*A. japonicus*) were obtained from the marine ranch of Laizhou Bay (37°18.382′N, 119°40.853′E) in China’s Bohai Sea. They were maintained in 1000 L aquariums containing aerated sand-filtered seawater (salinity 30‰, pH 8.0) and placed in a 12 h light (0700 h—1900 h)/12 h dark (1900 h—0700 h) condition for 2 weeks prior to use in the study. The water temperature and dissolved oxygen content were maintained at 16 ± 0.5°C and >6.0 mg/L, respectively. Sea cucumbers were fed an excess self-made diet (30% Sargassum powder, 70% sea mud) once a day at 0800 h. Before feeding, uneaten food and feces were siphoned carefully, then half of the tank water was exchanged to keep high water quality. Twenty four sea cucumbers (97.7 ± 14.8g) were selected to measure the concentration of melatonin in the coelomic fluid using a commercial melatonin ELISA kit (Zcibio, Shanghai, China). Before dissection, sea cucumbers were anesthetized using magnesium sulfate (0.4 M/L), which is considered to be an effective method with minimal negative effects on this species (Zhou et al., 2014). After each sea cucumber was dissected, 1 mL of coelomic fluid was pumped into a sterile tube, and tubes were stored in liquid nitrogen until use in the ELISA test. These samples were divided into eight groups (C1–C8) randomly and each three samples in one group were mixed together as one sample to determine the melatonin concentration based on the premise that an unknown amount of melatonin competes with a fixed amount of enzyme-labeled antigen for the binding sites of antibodies coated on the wells of the assay plate. Standards with precise concentrations were used in this detection procedure. Buffers, incubation times, shaking processes, and temperatures were used or set according to the kit instructions. Finally, absorbance was measured using a multifunctional microplate reader at 450 nm (Varioskan Flash, Thermo Scientific, Vantaa, Finland).

### Melatonin Administration and Locomotor Behavior Tests

After ELISA test, other 48 sea cucumbers were weighed and divided randomly into four groups (98.4 ± 16.2g, *n* = 12 sea cucumber/group) representing the three melatonin concentrations and the control. Melatonin (Solarbio Life Sciences, Beijing, China) was dissolved in 5% ethanol and diluted with sterile sand-filtered seawater to reach the required concentrations (10, 100, and 1000 μM). Sterile sand-filtered seawater with an equivalent amount of ethanol was used as the control. These experimental concentrations were set by reference to the relative study of melatonin affection on locomotor and feeding behavior (Pinillos et al., 2001; Azpeleta et al., 2010). The experiments were conducted in aquariums (50 cm × 50 cm × 50 cm), and the water line was maintained at 30 cm. A white acrylic board was placed at the bottom of each aquarium to make the sea cucumber easier to detect by the analysis software. An infrared camera (Hikvision, Hangzhou, China) fixed by plastic frames was installed above each aquarium. The photoperiod was identical to the condition of previous 2 weeks temporary cultivation. To maintain a soft and uniform light during the experiment, softboxes were placed around the aquariums, and all experimental facilities were enclosed by a black-out cloth. Before the locomotor behavior recording, sea cucumber individuals were placed in the test tank for at least 24 h to become familiar with the experimental environment. For the experiment, sea cucumbers were injected with 0.1% (v/w) (Kato et al., 2009) of a given melatonin concentration or the control via peripheral injection at 2200 h, so the zeitgeber time is 15 (ZT15) in our study. There are two reasons for why the injection made at this time point: (1). Sea cucumbers are very sensitive to lights (Dong et al., 2010; Sun et al., 2018), and their physiological status and activities are totally adjusted to dark at 3 h after turning to dark. (2). Previous study demonstrated that 2200 h is the beginning time of feeding peak (Sun et al., 2015), and the period of locomotor peak (0300–0600 h) of our preliminary study was almost the same as feeding peak (0200–0600 h). This design allowed an increase in circulating melatonin during the peak of activity in sea cucumber. The injections were performed with a 1 mL ICO syringe (JIANSHI^®^, Luohe, China) and a 0.3 mm Microlance needle, and they were done under dim red light conditions (Pinillos et al., 2001).

Locomotor activities were recorded by the infrared camera of Hikvision (DS-2CD3310D-I, 4MM, Hangzhou, China) for 12 h, and videos were analyzed using Ethovision (version 10.1) software (Noldus Inc., Netherlands). To evaluate the effect of melatonin on locomotor performance of the sea cucumbers, the following behaviors before and after melatonin injection were analyzed and counted: total distance traveled, cumulative duration of movement, mean and maximum velocity, total steps taken, mean and maximum stride (mean and maximum length of steps), and stride frequency (number of steps taken per minutes).

### Muscle Tissue Collection, UPLC-Q-TOF-MS Detection

The locomotor activity data were used to identify the time point and melatonin treating concentration at which a significant change in locomotor behavior occurred, and 24 sea cucumbers were collected at 7 h after administration and divided into 8 specimens averagely to acquire the tissues and measure the metabolites present in control (CON) and 1000 μM melatonin-treated (MEL) groups respectively. Sea cucumbers were anesthetized using magnesium sulfate (0.4 M/L). For each specimen, ∼2 g of longitudinal muscle tissue was carefully sheared off from the inner layer of the body wall and washed with ultrapure water. The samples were placed in a pipe and stored in a −80°C freezer prior to metabolomic analysis.

Before the extraction of metabolites, the samples from the −80°C freezer were stored for 30 min at −20°C and then thawed in a refrigerator at 4°C. For each specimen, 25 mg of muscle tissue were added to an Eppendorf tube containing 800 μl of ice-cold methanol and two small steel balls. All samples were ground using a TissueLyser (Tiangen, Beijing, China) at 50 Hz for 4 min. After grinding, the steel balls were removed, the samples were precipitated overnight at −20°C, and then they were centrifuged at 30000 × *g* for 20 min at 4°C. Finally, 550 μl of supernatant were collected from each sample and transferred to a new Eppendorf tube for subsequent UPLC-Q-TOF-MS analysis.

All samples were acquired by the LC-MS system following the manufacturer’s instructions. First, all chromatographic separations were performed using the UPLC system (Waters Corp., Manchester, United Kingdom). An ACQUITY UPLC BEH C18 column (100 mm × 2.1 mm, 1.7 μm, Waters) was used for the reversed phase separation. The column oven was maintained at 50°C. The flow rate was 0.4 mL/min, and the mobile phase consisted of solvent A (water + 0.1% formic acid) and solvent B (acetonitrile + 0.1% formic acid). Gradient elution conditions were set as follows: 0 – 2 min, 100% phase A; 2–11 min, 0–100% B; 11–13 min, 100% B; and 13–15 min, 0–100% A. The injection volume for each sample was 10 μl.

A high-resolution tandem mass spectrometer Xevo G2 XS QTOF (Waters) was used to detect metabolites eluted form the column. The Q-TOF was operated in both positive and negative ion modes. For positive ion mode, the capillary and sampling cone voltages were set at 3 kV and 40 V, respectively. For negative ion mode, the capillary and sampling cone voltages were set at 2 kV and 40 V, respectively. The MS data were acquired in Centroid MSE mode. The TOF mass range was 50–1200 Da, and the scan time was 0.2 s. For the MS/MS detection, all precursors were fragmented using 20 – 40 eV, and the scan time was 0.2 s. During the acquisition, the LE signal was acquired every 3 s to calibrate the mass accuracy. To evaluate the stability of the LC-MS during the whole acquisition, a quality control sample (pool of all samples) was acquired after every 10 samples.

### ATP and FFA Tests

The ATP levels in muscle tissues were measured using a luciferase-based enhanced ATP assay kit (S0027, Beyotime Biotechnology, Shanghai, China). In brief, the muscle tissues were fully dispersed, ground using a homogenate machine (BRS-200, BUNKIN, Anhui, China), and incubated in lysis buffer for 10 min. The mixtures were centrifuged for 5 min at 4°C and 12,000 × *g*, and each supernatant was collected in a new tube. Subsequently, 100 mL of detection solution were added to a 96-well plate and incubated for 5 min at room temperature. Next, 20 mL of each supernatant were added to the plate and mixed quickly with the detection solution. Luminescence was detected using the Varioskan Flash multifunctional microplate reader within 30 min. The concentration of ATP was calculated according to an ATP standard curve, and results were expressed as nmol/mg. Besides, a FFA assay kit was used to measure the FFA concentration in muscle tissues according to the kit instructions. FFA concentrations were calculated according to an FFA standard curve (y = 0.0038x + 0.0055, *R*^2^ = 0.994), and results were expressed as nmol/g.

### Statistical Analysis

Behavioral data acquired from video analysis and the ATP and FFA concentrations in muscle tissues were analyzed using one-way analysis of variance followed by Tukey’s *post hoc* multiple comparison tests. SPSS 20.0 software was used to conduct the analyses. A probability level of *p* < 0.05 was considered to be statistically significant.

The raw UPLC-Q-TOF-MS data were imported into Progenesis QI software to generate a data matrix that included retention time (RT), mass-to-charge ratio (m/z) values, and peak intensity. The main parameters were at default settings. After eliminating low-weight ions (Relative Standard Deviation > 30%), the quality control-based robust LOESS signal correction method was used to correct the data. To identify the key metabolites that differed between the CON and MEL groups, principal component analysis (PCA), partial least squares discriminant analysis (PLS-DA), fold-change (FC) analysis, and the *t*-test were performed on the UPLC-MS data. PCA is a descending dimension method in which multiple variables are transformed into a few key variables that can mirror the original overall variable information. PLS-DA is a multivariate statistical analysis method that is widely used in the analysis of metabolomics data. As a supervised analysis method, PLS-DA can provide an extremely comprehensive reflection of the distinction among the experimental samples (Boulesteix and Strimmer, 2007). In this model, parameter R2 denotes the explanation capacity and Q2 represents the predictive rate of the model. The variable importance of projection (VIP) values were calculated in the model. Potential metabolic biomarkers satisfy three conditions (VIP > 1, FC > 1.2/ < 0.8333, *q* < 0.05), and those that met these criteria were chosen as key metabolites with significant changes. Ultimately, significant metabolites were searched in the Kyoto Encyclopedia of Genes and Genomes biochemical databases to annotate related metabolic pathways.

## Results

### Existence of Melatonin in Coelomic Fluid of *A. japonicus*

Melatonin was present in the coelomic fluid of normal *A. japonicus* at concentrations ranging from 120.01 to 165.38 ng/L (Table 1). The average concentration was 135.68 ng/L.

**Table 1:** Concentrations of melatonin in the coelomic fluid of *A. japonicus* based on the method of ELISA test.

Sample ID	Optical density (OD)	Melatonin (ng/L)
C1	1.8563	120.84
C2	1.7844	132.25
C3	1.8616	120.01
C4	1.6396	156.86
C5	1.9784	123.95
C6	1.7245	142.14
C7	1.4843	165.38
C8	1.8360	124.02

### Effect of Melatonin on Locomotor Behavior of *A. japonicus*

**FIGURE 1:**
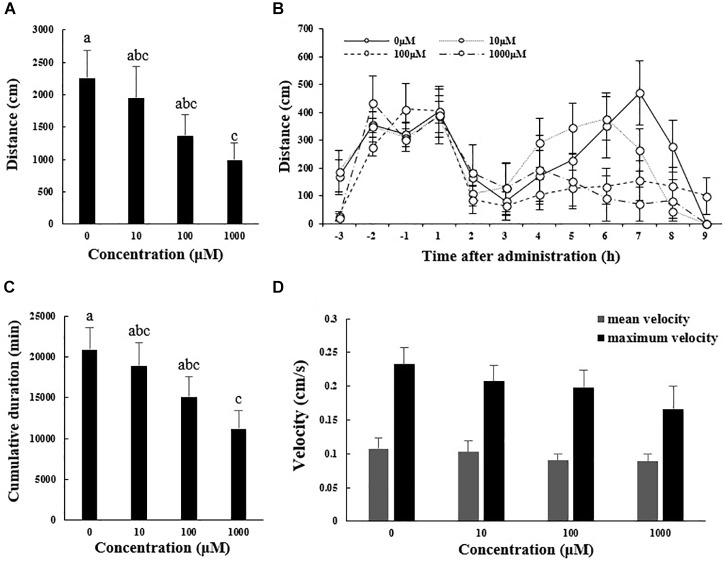
Total distance traveled **(A)**, cumulative duration of movement **(C)**, and mean and maximum velocity **(D)** for *A. japonicus* in the different treatment groups 9 h after intraperitoneal injection with melatonin or the control and distances moved within each hour interval before and after injection with melatonin or the control **(B)**. Results are shown as the mean ± SEM. Different letters indicate significant differences (*N* = 12, *p* < 0.05).

Figure 1 shows the locomotor performance (distance moved, cumulative duration of moving, and mean and maximum velocity) of *A. japonicus* in the experiment. The total distance moved by sea cucumber during the 9 h after melatonin administration significantly decreased with increasing melatonin dose, ranging from 22.53 ± 4.35 m in the control group to 9.93 ± 2.57 m in the 1000 μM melatonin group (Figure 1A; *F*_3,12_ = 12.828, *p* < 0.01). The distances moved within each hour interval before and after melatonin administration also decreased with increased melatonin concentration, and the maximum difference between control and treatments occurred at 7 h after injection (Figure 1B). Moreover, in the 100 and 1000 μM treatment groups, no typical peak point was observed after melatonin injection (Figure 1B). These results illustrate a declining trend in motility after melatonin administration, which explains the decreased cumulative duration of locomotion overall (Figure 1C; *F*_3,12_ = 13.101, *p* < 0.01). However, the velocity of motor behavior was low in *A. japonicus*. Although the mean and maximum velocities decreased to some extent with increased melatonin dose, the decreases were not statistically significant (Figure 1D; *F*_3,12_ = 7.865, *p* > 0.05).

**FIGURE 2:**
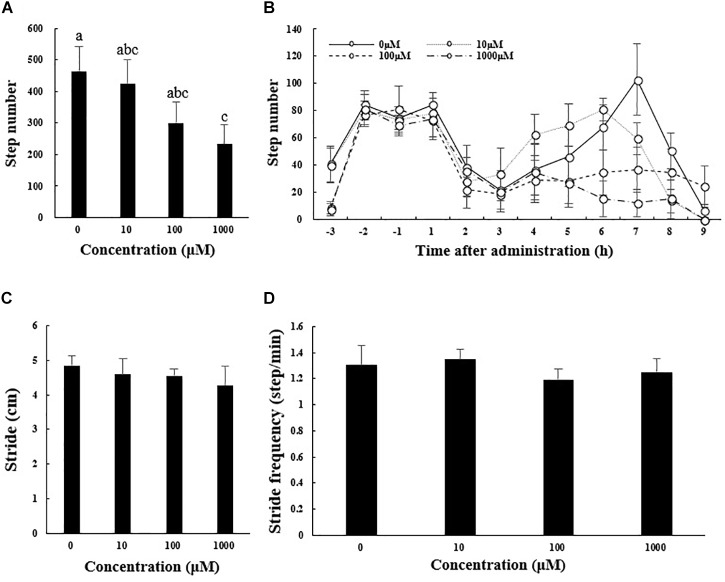
Total number of steps taken **(A)**, stride length **(C)**, and stride frequency **(D)** of *A. japonicus* in the different treatment groups 9 h after intraperitoneal injection with melatonin or the control and the number of steps taken within each hour interval before and after injection with melatonin or the control **(B)**. Results are shown as the mean ± SEM. Different letters indicate significant differences (*N* = 12, *p* < 0.05).

Figure 2 shows results for other measures of locomotor performance (number of steps taken, mean stride, and stride frequency). The total number of steps taken during 9 h after melatonin administration decreased from 465.17 ± 78.49 in the control group to 233.17 ± 62.14 m in the 1000 μM melatonin group (Figure 2A; *F*_3,12_ = 10.919, *p* < 0.01). The number of steps taken within each hour interval before and after melatonin injection showed a curve similar to that for distance traveled (Figure 2B). Mean stride and stride frequency decreased with increasing melatonin dose, but the differences were not statistically significant (Figure 2C,D).

### Effect of Melatonin on Muscle Physiology of *A. japonicus*

**FIGURE 3:**
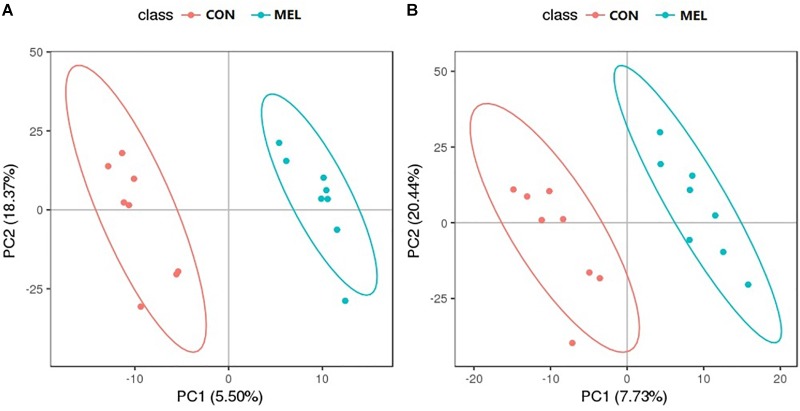
The partial least square discriminant analysis (PLS-DA) scores plot of muscle metabolites from the control (CON) and melatonin treated (MEL) groups in the positive ion **(A)** and negative ion **(B)** scan modes. The abscissa and ordinate represent the first principal component (PC1) and the second principal component (PC2) respectively.

**FIGURE 4:**
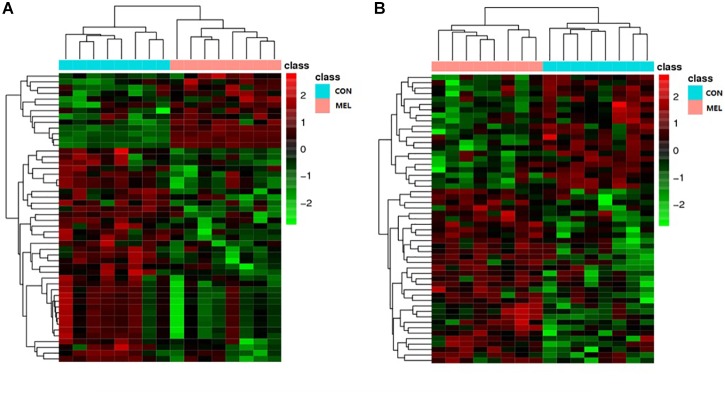
The heat maps of overall differential metabolites from CON and MEL groups in positive **(A)** and negative **(B)** mode. Each line represents a differential metabolite and each cross represents a muscle sample. Different colors represent different higher abundance intensity (mean value acquired from all detected samples of the same group).

The abscissa and ordinate in the PLS-DA plot represent the first principal component (PC1) and the second principal component (PC2), respectively (Figure 3). The percentages in parentheses indicate the proportion of the variance in the raw data explained by a given principle component. Thus, for the positive ion mode, 5.50 and 18.37% of the variance in the data could be explained by PC1 and PC2, respectively. In the negative ion mode, the values were 7.73% for PC1 and 20.44% for PC2. Each dot represents one sample. The CON (red) and MEL (blue) groups are clearly separated from each other in both ion modes. In addition, the heat maps of overall differential metabolites from CON and MEL groups in positive (A) and negative (B) mode are shown in Figure 4. These two figures indicate differences in the metabolites in the muscles of these two groups.

The *t*-test (*p* < 0.05) and PLS-DA model (VIP > 2.0) results together identified 22 metabolites from the positive and negative ion modes that differed between the CON and MEL groups (Table 2). These metabolites included (3S,6E)-nerolidol, serotonin, melatonin, flavin mononucleotide, retinoic acid, indole, and cinnamaldehyde. (3S,6E)-nerolidol and melatonin were significantly upregulated in the MEL group, whereas the other metabolites were downregulated. These metabolites are mainly involved in the peroxisome proliferator-activated receptor (PPAR) signaling pathway, oxidative phosphorylation, gap junction, arachidonic acid metabolism, protein digestion and absorption, and other metabolic pathways.

**Table 2:** Muscle metabolites with concentrations that differed significantly between the MEL and CON groups.

Metabolite	Ion mode	Mass (Da)	RT (min)	VIP value	FC_(MEL/CON)_	*p*
(3S,6E)-Nerolidol	M+K	233.1290	5.46	11.250	33.877	<0.01
Serotonin	M+H-H_2_O	159.0923	4.27	2.090	0.825	<0.05
Melatonin	M+H	233.1290	5.46	11.245	33.877	<0.01
2-Phenylacetamide	M+H- H_2_O	118.0660	4.27	2.157	0.807	<0.05
3-Methyldioxyindole	M+H- H_2_O	146.0608	4.27	2.260	0.807	<0.05
Cinnamaldehyde	M+H- H_2_O	115.0548	4.27	2.202	0.819	<0.01
Indole	M+H	118.0660	4.27	2.160	0.807	<0.05
Indoleacetaldehyde	M+H- H_2_O, M+H	159.0691	4.27	2.179	0.814	<0.05
Tryptophanol	M+H- H_2_O	144.0813	4.27	2.200	0.811	<0.05
Normorphine	M+H- H_2_O	254.1185	4.05	2.512	0.757	<0.05
2-Propylglutaric acid	M+NH_4_	192.1238	0.65	2.520	0.757	<0.05
Atropaldehyde	M+H- H_2_O	115.0548	4.27	2.202	0.819	<0.01
(S)-3-Hydroxy-*N*-methylcoclaurine	M+Na	338.1358	4.09	2.569	0.704	<0.05
5-KETE	M-H	317.2110	8.47	2.398	0.673	<0.05
12-KETE	M-H	317.2110	8.47	2.398	0.673	<0.05
15-KETE	M-H	317.2110	8.47	2.398	0.673	<0.05
Flavin Mononucleotide	M-H	455.0964	4.74	2.070	0.668	<0.05
Leukotriene A4	M-H	317.2110	8.47	2.400	0.673	<0.05
All-*trans* retinoic acid	M-H	299.2006	8.47	2.150	0.730	<0.05
9-*cis* retinoic acid	M-H	299.2006	8.47	2.150	0.730	<0.05
4-Oxoretinol	M-H	299.2006	8.47	2.153	0.730	<0.05
9,13-cis-Retinoate	M-H	299.2006	8.47	2.153	0.730	<0.05

### FFA and ATP Content in the Muscle of *A. japonicus*

To investigate the condition of the energy supply in muscles of sea cucumbers in the CON and MEL groups, the concentrations of FFA and ATP in muscle tissue were measured (Table 3). The concentration of ATP in the muscles of sea cucumbers after melatonin administration was significantly lower than that in the control group (*p* < 0.05), whereas the FFA concentration in the MEL group was higher than that in the CON group, although the difference was not statistically significant.

**Table 3:** Concentrations of FFA and ATP in the muscle tissue of sea cucumbers in the MEL and CON groups.

Group	FFA (nmol/g)	ATP (nmol/mg)
CON	125.25 ± 17.64^a^	8.85 ± 0.34^a^
MEL	146.47 ± 19.17^a^	5.22 ± 0.28^b^

*Different letters indicate significant differences at *p* < 0.05 (mean ± SE). FFA and ATP represent the concentrations of FFA and ATP in muscle tissue of sea cucumber respectively.*

## Discussion

### Existence of Melatonin in *A. japonicus*

Our study demonstrated the existence of melatonin in the coelomic fluid of *A. japonicus*. Many types of coelomocytes are suspended in the coelomic fluid, and they are involved in immunity, trephocytic activities, and nutrient transport (Eliseikina and Magarlamov, 2002). Considering the function of coelomic fluid in the biological system of the sea cucumber, it is reasonable to speculate that melatonin could be present in many other tissues or organs, such as the intestine, gonad, and body wall. To the best of our knowledge, only a few studies have investigated melatonin in echinoderms, despite their important phylogenetic and ecological status. Endogenous melatonin was detected in the gonad of the sea star *Echinaster brasiliensis*, and its production peaked at night (Peres et al., 2014). The enzymes arylalkylamine *N*-acetyltransferase and tryptophan hydroxylase were proposed as possible regulators of the production of melatonin in this species (Peres et al., 2014). In addition, three melatonin receptor sequences were identified from the genome of the sea urchin *Strongylocentrotus purpuratus* (D’Aniello et al., 2015). Therefore, we hypothesize that melatonin is a common molecule in echinoderms, which further supports the premise that melatonin is a phylogenetically ubiquitous molecule in organisms with a deep evolutionary origin (Poeggeler et al., 1991).

### Effect of Melatonin on Locomotor Performance of *A. japonicus*

Our results strongly support the premise that melatonin is involved in regulation of locomotor activities in *A. japonicus*, particularly locomotor endurance. The decrease of total distance traveled, total steps taken, and cumulative duration of movement with increasing dosage of melatonin suggests that melatonin can greatly reduce the endurance of movement in this species. The effect of melatonin on locomotor activity depends on the location of administration, time of administration, and circadian rhythms (diurnal or nocturnal) (López-Olmeda et al., 2006; Azpeleta et al., 2010). Melatonin decreased the locomotor endurance in scotophase and eliminated the locomotor activity cycles in the crayfish *Procambarus clarkii* (Tilden et al., 2003a) and the fiddler crab *Uca pugilator* (Tilden et al., 2003b). Although the locomotor circadian rhythm of sea cucumbers is still unclear, considering the administration time point, our results are consistent with results of studies of other invertebrates. Decreased locomotion has also been widely observed after exogenous application of melatonin in vertebrate species, including fishes (Zhdanova et al., 2001; López-Olmeda et al., 2006; Azpeleta et al., 2010), reptiles (Hyde and Underwood, 2000), birds (Murakami et al., 2001), mammals (Zhdanova, 2005), and platyhelminthes (Omond et al., 2017). These results all support the idea that the administration of melatonin caused sleep-like behavior in diverse species.

In addition, the average and maximum velocity, mean stride length, and stride frequency of *A. japonicus* in the control group were higher than those in the treatment group, indicating that melatonin can decrease the movement efficiency of this species. This result coincides with a previous result reported for an invertebrate, the nematode *Caenorhabditis elegans*. The locomotion rates of *C. elegans* significantly decreased after administration of exogenous melatonin (Tanaka et al., 2007). By means of genetics and mutant strains, melatonin acted as a direct neuromodulator by regulating locomotor activity in *C. elegans* (Tanaka et al., 2007).

In summary, results of the present study indicate that melatonin diminished locomotor behavior—more precisely, locomotor endurance and efficiency—in *A. japonicus*. This finding provides evidence for the effect of melatonin on the locomotor behavior of sea cucumbers.

### Potential Mechanisms Involved in the Decrease of Locomotor Performance of *A. japonicus* After Melatonin Administration

To date, most studies of the effect of melatonin on locomotor behavior have focused on the alteration of behavior, and little attention has been paid to the potential physiological changes involved in locomotor influence by melatonin. In present study, we selected the time point at which we observed maximum differentiation in locomotor performance between control and melatonin-treated sea cucumbers (7 h after melatonin administration) and measured metabolite levels in organisms each group. Treated sea cucumbers and control sea cucumbers had different muscle metabolite profiles, and it was clear that a combination rather than a single metabolic pathway resulted in the loss of locomotor performance. In addition to higher levels of melatonin in treated animals, which probably was due to the exogenous melatonin administration, serotonin, flavin mononucleotide, 9-cis-retinoic acid, and all-tans retinoic acid were downregulated. These metabolites are categorized into four main pathways: metabolic, gap junction, oxidative phosphorylation, and PPAR signaling. These metabolites and the pathways in which they participate suggest a potential mechanism by which melatonin caused the decreased locomotor performance in *A. japonicus*.

Serotonin is an indole derivate that is widely distributed in the tissues of animals and plants (Nässel, 1988; Murch et al., 2001). It is speculated to be one of the most critical, but poorly understood transmitters involved in many brain functions, such as mood, eating, and sleep, as well as depression and some other psychiatric disorders (Uchida and Cohen, 2017). In echinoderms, serotonin is demonstrated to be contained in the oocytes, zygotes, blastomeres and adults of starfishes (Squires et al., 2010). Previous studies have also reported the distribution and concentrations of serotonin and serotonin-like compounds in the embryos of sea urchins (Renaud et al., 1983; Morikawa et al., 2001; Shmukler and Tosti, 2001; Buznikov et al., 2005). Furthermore, serotonin has mainly been identified biochemically in the central nervous system of many invertebrates (Welsh and Moorhead, 1960; Fujii and Takeda, 1988), where it is a neurotransmitter that acts as a messenger in the “communication” of the nervous system (Nässel, 1988). A number of studies revealed that serotonin stimulates or triggers the locomotor behavior of animals, including rats (Cabaj et al., 2017), fish (Byrne, 1970), mollusks (Pavlova, 2001), and marine trematodes (Tolstenkov et al., 2017). At the cellular and behavioral levels, serotonin is considered to be a powerful modulator rather than a mediator that takes part in vertebrate motor function (Jacobs and Fornal, 1997; Feraboli-Lohnherr et al., 1999). In *C. elegans*, the motor circuits in chemical synapses, gap junctions, and neuromuscular junctions are mediated by serotonin and dopamine, and this is the pathway that *C. elegans* uses to modulate motor behavior (Sawin et al., 2000; Cao et al., 2005; Chase and Koelle, 2007). Consumption of serotonin by the central nervous system resulted in increased locomotor activity in a number of mammals, possibly due to the direct effect of serotonin on the locomotor system (Jacobs and Fornal, 1997). In contrast, systematic melatonin administration restrained the release of serotonin in the brain and led to a reduction of locomotor performance in rats (Chuang and Lin, 1994). This effect of melatonin on motor behavior agrees with our results for *A. japonicus*. In addition, the study of locomotion in snails *Helix lucorum* revealed that the length of pedal waves (stride) increased after serotonin injection (Pavlova, 2001). Although the difference was not significant, the stride length in the melatonin-treated group of *A. japonicus* decreased with increasing melatonin dose, which would occur as a result of reduced serotonin level. In our study, metabolomic results revealed a significant reduction of serotonin in muscles after melatonin administration. Based on the depressed locomotor performance of sea cucumbers in the melatonin-treated group and the known modulatory role of serotonin in the locomotor system, we suggest that downregulation of serotonin was an important contributor to the decreased locomotor performance observed in melatonin-treated sea cucumbers.

There is an interrelationship between melatonin and serotonin systems (Gaffori and Van Ree, 1985) and serotonin is involved in the synthesis of melatonin (Sprenger et al., 1999). The levels of serotonin are high in the daytime and decreased at night, which is contrary to the rhythm of melatonin (Ganguly et al., 2002). Treating with melatonin caused a dose-dependent reducing of serotonin release (Chuang et al., 1993). Injection of serotonin antagonists (methysergide and cyproheptadine) gave rise to decreased locomotor activity as was found after administration of melatonin (Gaffori and Van Ree, 1985). The melatonin production pathway in starfish *E. brasiliensis* is likely to be the same as previously described in vertebrates by enzymatic assays (Peres et al., 2014). The increase of arylalkylamine *N*-acetyltransferase (AANAT) activity reduces the nocturnal serotonin levels and results in an increase in the intracellular concentration of *N*-acetylserotonin (NAS), which will convert to melatonin by hydroxyindole-*O*-methyltransferase (HIOMT) (Ganguly et al., 2002). In addition, AANAT activity can be finely modulated and decreases sharply when sympathetic stimulation is ceased by treating with adrenergic antagonists or nocturnal photostimulation (Deguchi and Axelrod, 1972; Klein and Weller, 1972; Parfitt et al., 1976). Hence, the depression of melatonin on serotonin is likely to be mediated by AANAT activity. However, melatonin is highly pleiotropic (Hardeland et al., 2006), which can also be demonstrated by our results of metabolomic data, and even the gene of AANAT was not blasted from the genome of *A. japonicus*, which suggest a special relationship between melatonin and serotonin in this species. Therefore, internal mechanisms for the decrease of serotonin after melatonin administration still need further studies, especially in *A. japonicus*.

9-*cis*-retinoic acid (9cRA) and all-*trans* retinoic acid (atRA) are all bioactive retinoids that belong to vitamin A metabolites (Heine et al., 2018). 9cRA is generated from atRA by spontaneous isomerization *in vivo* (Yu et al., 2017; Heine et al., 2018) and they can regulate metabolism by activating two specific nuclear receptors——retinoic acid receptor (RAR) and retinoic X receptor (RXR) (Egea et al., 2000; Ziouzenkova and Plutzky, 2008; Abed et al., 2017). Interestingly, RARs can bind both atRA and 9cRA, whereas RXRs exclusively binds 9cRA (Heyman et al., 1992; Egea et al., 2000; Abed et al., 2017). Retinoic acid signaling is relayed through these two nuclear receptors (Viera-Vera and García-Arrarás, 2018). For a long time, this signaling was regarded as a chordate innovation, the presence and function of it in invertebrates are therefore rarely studied (Gutierrez-Mazariegos et al., 2014). In echinoderms, RAR have ever been identified in sea urchins. Besides, the sequences of RAR and RXR as well as their isoforms are recently identified and characterized in the sea cucumber *Holothuria glaberrima*, and both retinoid receptors are expressed in all sampled tissues including muscles (Viera-Vera and García-Arrarás, 2018). Moreover, retinoic acid signaling is also existed in the starfish *Patiria pectinifera*, and serves as a regulator in metamorphosis process (Yamakawa et al., 2018). As an obligate heterodimeric partner for other nuclear receptors, including PPARs (α, δ/β, and γ), which are ligand-activated transcription factors that regulate gene expression (Ziouzenkova et al., 2002), RXR helps coordinate energy balance (Ziouzenkova and Plutzky, 2008). PPARα heterodimerizes with RXRβ, and they cooperate to activate the acyl-CoA oxidase gene promoter (Keller et al., 1993). Due to the rate-limiting enzyme of the peroxisomal β-oxidation pathway of fatty acids is encoded by the acyl-CoA oxidase gene, PPARs and RXRs play an important role in lipid metabolism (Dreyer et al., 1992; Tugwood et al., 1992; Keller et al., 1993). Since the presence of PPARs and RXRs in echinoderms (Pagliarani et al., 2012; Viera-Vera and García-Arrarás, 2018; Yamakawa et al., 2018), this process of lipid metabolism regulation is likely to be existed in sea cucumbers. According to our result, 9cRA and atRA were downregulated in the muscles of melatonin-treated sea cucumbers. The reduced 9cRA and atRA levels may decreased the formation of ligand/receptor complex of PPARs and RXR, downregulated their ability to activate the acyl-CoA oxidase gene, and thereby inhibited the peroxisomal β-oxidation pathway of fatty acids. In addition, the relatively high levels of FFAs may also indicate insufficient fatty acid consumption in the melatonin-treated groups. Because of the energy supply function of fatty acids, inadequate energy supply for muscles to contract for locomotion is predicted in sea cucumbers after melatonin administration.

Flavin mononucleotide (riboflavin-5-phospate, FMN) is an important organic cofactor in living cells and plays a vital role in biological systems (Yan et al., 2018). To be more precise, FMN is a coenzyme for flavoprotein, which can pass electrons and hydrogen atoms and is involved in the mitochondrial oxidative phosphorylation process in animals (Zheng et al., 1990; Sakai and Takahashi, 1996). It is well-know that the mitochondrial oxidative phosphorylation system is the final biochemical pathway that takes part in the production of ATP——the energy substrate for muscle contraction (Mommaerts, 1969; Smeitink et al., 2001). Actually, mitochondria generate most of the energy in cells needed for animal activity primarily through the oxidative phosphorylation process, in which electrons are passed along a series of respiratory enzyme complexes that are located in the inner mitochondrial membrane (Gibson and Hastings, 1962; Saraste, 1999). The energy produced by electron transfer is devoted to pumping protons across membrane, and then the electrochemical gradient produced from this process enables another complex, ATP synthase, to synthesize the energy carrier ATP (Saraste, 1999). In the melatonin-treated group of sea cucumbers in our study, FMN was significantly downregulated, and this would have had a negative effect on electron transfer, inhibited protons from crossing the membrane, and reduced the production of ATP in muscle. Locomotor behavior of sea cucumbers involves a relatively simple alternating pattern of contraction and extension of the body wall, which is under the control of muscle (Qiu et al., 2014). While ATP plays a fundamental role in supplying energy for muscle contraction (Saraste, 1999). We therefore suggest that this effect combined with the actual decrease of ATP in the muscles of sea cucumbers in the melatonin-treated group decreased the ATP supply for muscle contraction by oxidative phosphorylation and resulted in insufficient energy to support locomotor activity. Consequently, the hypodynamic muscles along the body wall of the sea cucumbers led to the significant decrease in the locomotor ability of treated sea cucumbers compared to control animals.

## Conclusion

In this study, we detected melatonin in the coelomic fluid of *A. japonicus* and showed that melatonin had a sedative effect on this species. Locomotor endurance, including movement distance, number of steps taken, and cumulative duration of movement, significantly decreased with increasing dose of melatonin. Locomotor efficiency (e.g., velocity, stride length, and stride frequency) also decreased to some extent with increasing dose of melatonin. The metabolomic results combined with FFA and ATP data indicated that there are a number of potential physiological mechanisms that can explain the inhibitory effect of melatonin on locomotor endurance and efficiency of *A. japonicus*. These include reduction of the locomotor modulator — serotonin, inhibited fatty acid oxidation and disturbed oxidative phosphorylation.

## Author Contributions

KD and LZ designed and performed the research. TZ and HY contributed new reagents and analytic tools. KD analyzed the data and wrote the manuscript. LZ and HY supervised the research. RB revised this manuscript.

## Conflict of Interest Statement

The authors declare that the research was conducted in the absence of any commercial or financial relationships that could be construed as a potential conflict of interest.
